# Dysregulation of the Skin–Liver Axis in Prurigo Nodularis: An Integrated Genomic, Transcriptomic, and Population-Based Analysis

**DOI:** 10.3390/genes15020146

**Published:** 2024-01-23

**Authors:** Melika Marani, Vrinda Madan, Thomas K. Le, Junwen Deng, Kevin K. Lee, Emily Z. Ma, Shawn G. Kwatra

**Affiliations:** Department of Dermatology, Johns Hopkins University School of Medicine, Baltimore, MD 21205, USA

**Keywords:** prurigo nodularis, itch, pruritus, hepatic, liver, comorbidity, genomic, transcriptomic, epidemiology

## Abstract

Pruritus has long been linked to hepatic dysfunction; however, there are limited data characterizing the association between liver disease and prurigo nodularis (PN), a chronic inflammatory skin disease featuring severe pruritis. We thus conducted a cross-sectional analysis of hepatic comorbidities in PN patients using TriNetX, a large global health research network. This analysis revealed that PN patients had a higher risk (*p* < 0.001) of developing liver cirrhosis, acute and subacute hepatic failure, inflammatory liver disease, chronic hepatitis, nonalcoholic steatohepatitis, portal hypertension, fatty liver, chronic passive congestion of the liver, and hepatocellular carcinoma compared with healthy controls. The cumulative incidence of liver disease was about three times higher in PN patients compared with healthy controls. These findings provided the basis for translational studies to investigate a genetic mechanism for this association. Cutaneous transcriptomic analysis performed on PN patients revealed the dysregulation of genes related to hepatic failure in lesional PN compared with both nonlesional PN and control skin. Similarly, gene set variation analysis (GSVA) revealed a significantly increased (*p* < 0.05) activation of liver metabolism, chronic hepatic failure, acute hepatic failure, cholestatic liver disease, polycystic liver disease, and hepatocellular carcinoma pathways in lesional PN compared with control skin. A subsequent genome-wide association study (GWAS) identified shared single-nucleotide polymorphisms (SNPs) in the genes *AR*, *EDIL3*, *MACROD2*, *PCSK5*, *RUNX1T1*, *TENM4*, and *ZEB2* between PN and liver disease from the FinnGen cohort. Significant dysregulation of the skin–liver axis in PN patients may explain the increased incidence and severity of hepatic comorbidities and help identify future therapeutic targets for PN.

## 1. Introduction

Prurigo nodularis (PN) is a chronic inflammatory skin disease presenting with intensely pruritic, symmetrically distributed hyperkeratotic nodules that favor the trunk and extremities [1]. The hallmark of PN is severe and unrelenting pruritus, resulting in a dramatic reduction in quality of life even when compared with other pruritic dermatoses such as atopic dermatitis and psoriasis [2,3]. PN predominantly affects middle- to old-aged adults, with a greater frequency and intensity of disease observed in females [4,5]. Additionally, PN disproportionately affects African Americans and is significantly associated with various systemic, cardiovascular, and psychiatric comorbidities [5,6,7,8]. These include heart failure, diabetes, depression, malignancy, infectious disease, chronic renal failure, HIV, and liver disease [9,10,11].

Of these many systemic associations and potential etiologies of PN, liver disease has been highly implicated with chronic pruritus [12,13,14,15]. The prevalence of pruritis in chronic liver disease has been reported to be 53%, with an additional increase to 70% in patients with primary biliary cholangitis [16,17]. Additionally, there have been growing reports of a skin–liver axis mediating liver-derived systemic inflammation and dermatological diseases [18]. Although pruritus is a significant comorbidity associated with liver disease, there are limited data characterizing the specific relationship of PN and hepatic complications. Given that pruritus with or without prurigo nodules is associated with underlying genetic susceptibility, we hypothesized that there is mechanistic disease linkage between liver pathologies and PN [19]. We tested this hypothesis by performing a population-level analysis to identify liver comorbidities of PN and by RNA sequencing analyses from the skin of patients with PN and healthy controls.

## 2. Materials and Methods

### 2.1. Population-Level Analysis

We used TriNetX, a global healthcare network that aggregates de-identified electronic medical records from approximately 73 million patients across several international healthcare organizations in a self-updating platform. Patients with PN were identified as having at least 2 instances of an International Classification of Diseases, Tenth Revision, Clinical Modification (ICD-10-CM) code for PN (ICD-10 L28.1) and no diagnosis of AD (ICD-10 L20.9) [8]. Similarly, patients with AD were identified as having at least 2 instances of an ICD-10 code for AD and no diagnosis of PN. Patients who lacked any diagnosis of dermatitis (ICD-10 L20–L30) were included as controls. In the relative risk analysis, patients with PN and AD were matched to healthy controls based on age, sex, race, and BMI using 1:1 propensity score matching. Subjects ≥ 18 years old from 2015 to 2022 were included. Hepatic comorbidities were identified by the presence of corresponding ICD-10-CM codes for each disease.

### 2.2. Skin RNA Sequencing

Skin biopsies were collected prospectively from patients with PN and sex-, age- (±5 years), and ethnicity-matched healthy control patients as has been previously described [20]. Inclusion criteria for patients with PN were diagnosis by a board-certified dermatologist and the presence of at least 20 nodules on more than one extremity [21]. Adult patients with clinically healthy, non-pruritic skin were included as controls. Patients with a known history of cutaneous or atopic diseases such as AD or psoriasis were excluded from the study. Informed consent was obtained from each participant. This study was approved by the Johns Hopkins Institutional Review Board (IRB00119007).

Lesional skin biopsies were obtained from the most pruritic nodules on the extremities or trunk. Control biopsies were obtained from matched locations in healthy patients. Nonlesional biopsies were obtained from normal-appearing skin ≥ 10 cm from the lesional biopsy site. After total RNA was extracted from biopsies, RNA-seq libraries were prepared using the KAPA Stranded mRNA-Seq Kit. The Illumina NovaSeq 6000 sequencer was then used to perform sequencing. The fastp toolkit was used to trim low-quality bases, and RNA sequencing data were further processed using Illumina sequencing adapters from the 3′ ends of the reads. The STAR RNA-seq alignment tool was used to map reads to the GRCh38v93 version of the human genome and transcriptome. Gene counts were compiled with the feature Counts tool. Differential gene expression was calculated using the DESeq2 R Bioconductor package. Differentially expressed genes were defined as those with less than or greater than a 1.5 log2-fold change.

### 2.3. Statistical Analysis

All statistical analyses were performed in R version 4.0.3. In the population-level relative risk analysis, PN patients were matched to control patients by adjusting for age, sex, race, and BMI using 1:1 propensity score matching. This was conducted to increase external validity and minimize confounding. Risk ratios and 95% confidence intervals of hepatic comorbidities were calculated. *p*-values were adjusted for multiple hypotheses using the Benjamini–Hochberg method. Hepatic-failure-related dysregulation of gene sets was assessed using the GSVA R Bioconductor package [22].

IPA of gene expression identified top toxicity pathways in PN lesions. Differentially expressed genes were calculated and the DESeq2 Bioconductor package for R was used to conduct the normalization and differential expression of genes. The false discovery rate was calculated to control for multiple hypothesis testing.

### 2.4. Disease Associations in FinnGen

The FinnGen database consists of genotype data from Finnish biobanks and phenotype data from digital health records in Finnish health registries (https://www.finngen.fi/en (accessed on 16 September 2021)). Informed consent was obtained from patients and healthy control participants in the FinnGen cohort under protocols approved by the local research ethics committees based on the Finnish Biobank Act. The FinnGen study is approved by the Finnish Institute for Health and Welfare (THL) approval number THL/2031/6.02.00/2017 (amendments THL/1101/5.05.00/2017, THL/341/6.02.00/2018, THL/2222/6.02.00/2018, THL/283/6.02.00/2019), the Digital and Population Data Service Agency VRK43431/2017-3, VRK/6909/2018-3, the Social Insurance Institution (KELA) KELA 58/522/2017, KELA 131/522/2018, KELA 70/522/2019, and Statistics Finland TK-53-1041-17. FinnGen samples were genotyped using Illumina and Affymetrix arrays (Illumina, Inc., San Diego, CA, USA; Thermo Fisher Scientific, Santa Clara, CA, USA). Genotypes were imputed using the Finnish-population-specific SISu v3 reference panel with Beagle 4.1 (version 08Jun17.d8b). Details of the genotype imputation workflow are described in the following protocol: https://dx.doi.org/10.17504/protocols.io.xbgfijw (accessed on 16 September 2021).

The available SNPs were searched from FinnGen genotypes “prurigo nodularis” and “diseases of liver”, and were screened for variants with a *p*-value of <0.05. Tissue and gene-based analyses were performed using the GENE2FUNC tool in FUMA [23]. To identify overlapping single-nucleotide polymorphisms (SNPs) between PN and liver disease, we selected all shared genes with a log2 transcript count per million (TPM) normalized gene expression of ≥1 in skin from the GTEx project v8 [24]. Genes that had a log2 TPM of ≥1 in the pancreas were excluded to control for genes that are constitutively expressed in multiple organs.

## 3. Results

### 3.1. Population-Level Analysis of PN

**Figure 1:**
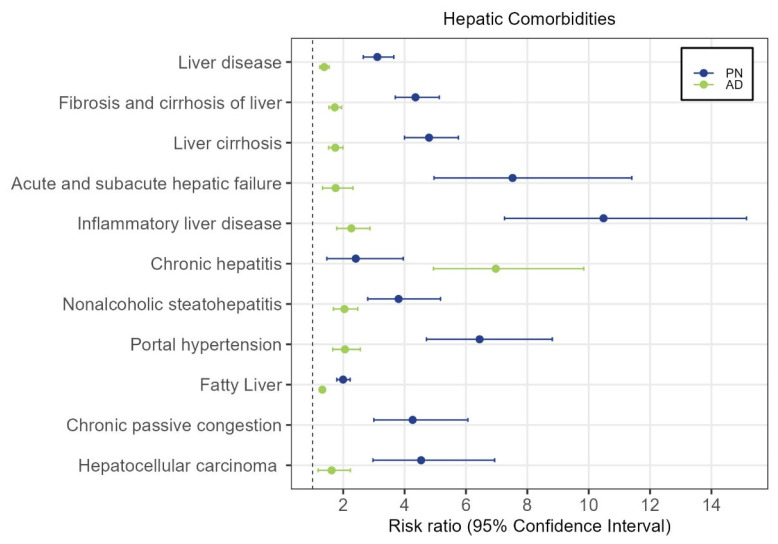
Hepatic comorbidities in patients with prurigo nodularis (PN) and atopic dermatitis (AD). Patients with PN and AD were compared with control patients. The listed hepatic comorbidities correspond to the ICD-10 codes utilized in clinical practice.

**Figure 2:**
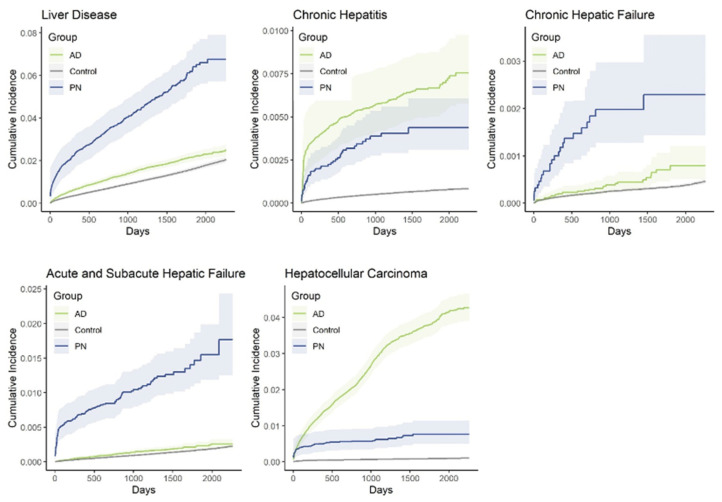
Cumulative incidence of liver disease and comorbidities in patients with prurigo nodularis (PN) and atopic dermatitis (AD).

In total, 15,911 PN patients were identified, with the mean age being 59.8, and 58% being female. In the AD cohort, 71,253 patients were identified; the mean age was 43.5 and 62% were female. Propensity-score-matched controls had identical age and sex distributions. Compared with controls, both PN and AD were associated with a higher risk ratio of developing liver disease, liver fibrosis and cirrhosis, acute and subacute hepatic failure, inflammatory liver disease, chronic hepatitis, nonalcoholic steatohepatitis, portal hypertension, fatty liver, and hepatocellular carcinoma (HCC), but the risk was higher in PN compared with AD for all diseases except chronic hepatitis (*p* < 0.001, Figure 1). Chronic passive congestion was also uniquely associated with PN and not AD. The overall cumulative incidence of liver disease among PN patients was over twice as high as it was in AD patients, and over three times as high as it was in control patients after 6.4 years (PN: 6.76%, CI 5.73–7.90% vs. AD: 2.90%, CI 2.20–3.76% vs. control: 2.16%, CI 2.01–2.32%, Figure 2). The cumulative incidence of chronic hepatic failure and acute and subacute hepatic failure was similarly higher in PN compared with AD and controls. However, the cumulative incidences of chronic hepatitis and hepatocellular carcinoma were higher in AD compared with PN.

### 3.2. Cutaneous Expression of Hepatic Failure-Related Genes in PN

**Figure 3:**
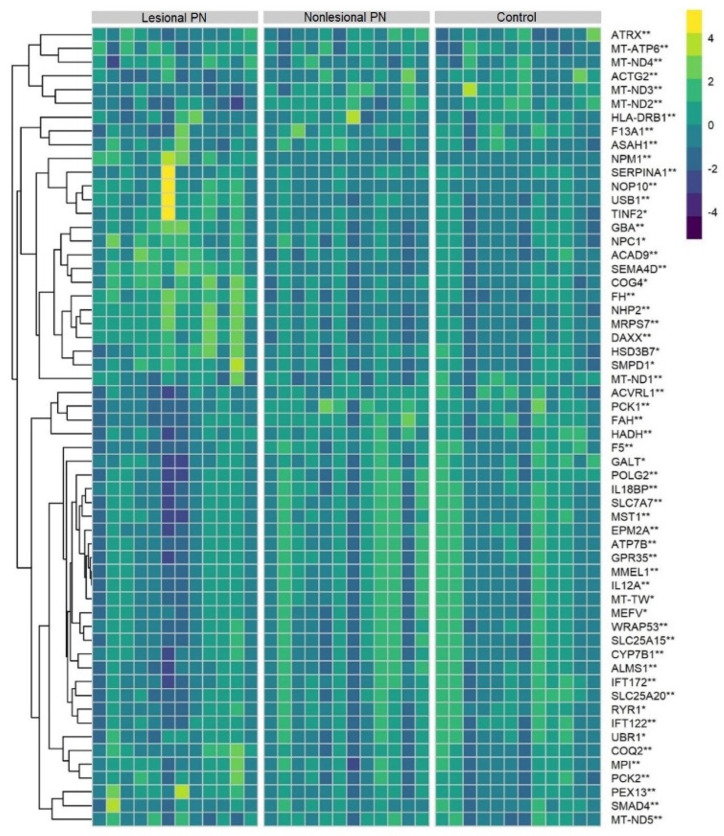
Heatmap of cutaneous mRNA expression of hepatic-failure-related genes in the skin of prurigo nodularis patients and healthy controls. Yellow, greater expression; purple, lower expression. ∗ Dysregulated in lesional vs. control. ∗∗ Dysregulated in both lesional vs. nonlesional and lesional vs. control.

The mean age of PN patients was 54.3  ±  14.1 years, 85% were female, and 77% were Black, with identical distributions for matched controls. Dysregulation of hepatic-failure-related genes was found in both lesional and nonlesional PN skin, shown as a heatmap in Figure 3.

**Figure 4:**
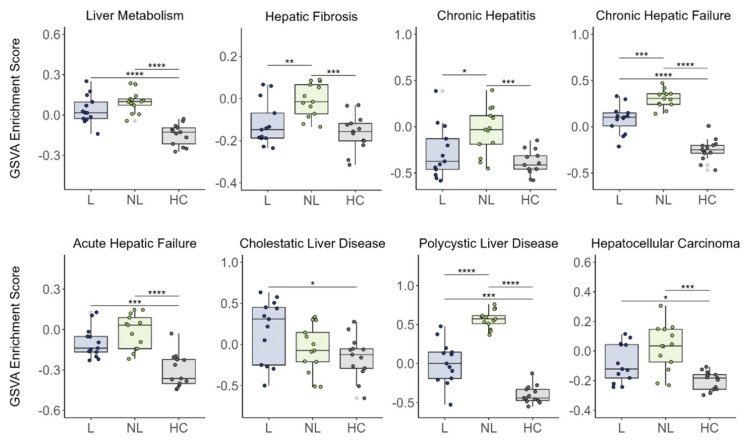
Gene set variation analysis of liver-disease-related pathways in lesional (L) PN (prurigo nodularis, *n* = 13), nonlesional (NL) PN (*n* = 13), and healthy control (HC, *n* = 13) skin. ∗ *p* < 0.05, ∗∗ *p* < 0.01, ∗∗∗ *p* < 0.001, and ∗∗∗∗ *p* < 0.001.

GSVA revealed the increased activation of several liver-disease-related pathways in PN patients compared with controls (Figure 4). Lesional PN skin had a significantly increased activation of liver metabolism (*p* < 0.0001), chronic hepatic failure (*p* < 0.0001), acute hepatic failure (*p* < 0.001), cholestatic liver disease (*p* < 0.05), polycystic liver disease (*p* < 0.001), and hepatocellular carcinoma (*p* < 0.05) compared with control skin. Nonlesional PN skin had a significantly increased activation of liver metabolism (*p* < 0.0001), hepatic fibrosis (*p* < 0.001), chronic hepatitis (*p* < 0.001), chronic hepatic failure (*p* < 0.0001), acute hepatic failure (*p* < 0.0001), polycystic liver disease (*p* < 0.0001), and HCC (*p* < 0.001) compared with control skin.

**Figure 5:**
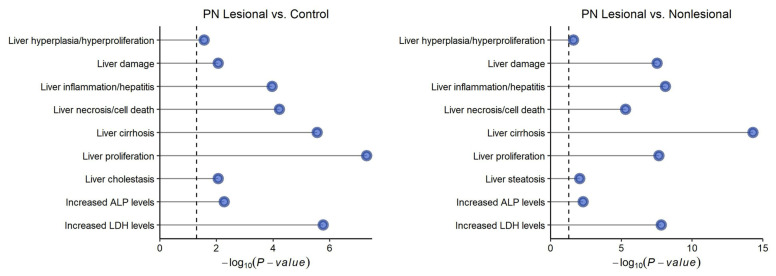
Significant hepatic toxicity pathways in the skin of patients with prurigo nodularis (PN), determined by ingenuity pathway analysis. Dotted line represents *p* = 0.05.

With ingenuity pathway analysis (IPA), the most significant hepatic toxicity pathways in PN lesions were liver hyperplasia/proliferation, liver damage, liver cholestasis, liver inflammation/hepatitis, liver necrosis/cell death, liver cirrhosis, liver proliferation, increased ALP levels, and increased LDH levels (Figure 5).

### 3.3. Genome-Wide Association Analysis for PN and Liver Disease

**Figure 6:**
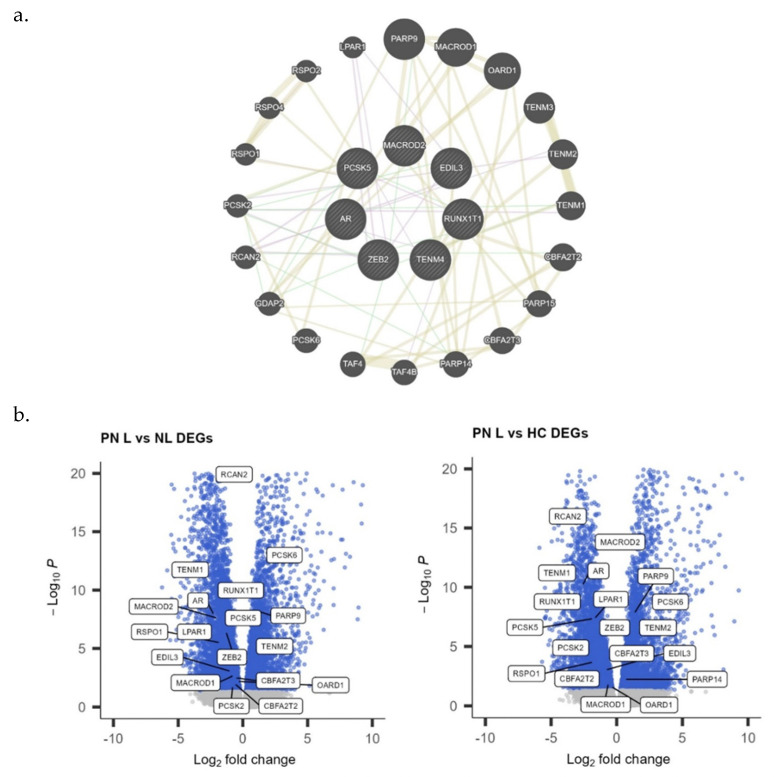
(**a**) GeneMANIA functional association gene network for the nearest genes from single-nucleotide polymorphisms expressed in skin and present in both prurigo nodularis and liver disease genome-wide associations. The coexpression and physical interaction between the genes are expressed as purple and green lines, respectively. (**b**) Differentially expressed genes (DEGs) from the GeneMANIA gene network in lesional vs. nonlesional (L vs. NL) and lesional vs. healthy control (L vs. HC) skin of patients with prurigo nodularis.

To characterize the association between PN and hepatic disease using a broader cohort of patients, GWAS was conducted to identify overlapping SNPs present in the skin between the two disease processes. The nearest genes from these shared SNPs included *AR*, *EDIL3*, *MACROD2*, *PCSK5*, *RUNX1T1*, *TENM4*, and *ZEB2* (Figure 6a). GeneMANIA was used to create a functional association gene network and identify other related genes. Differentially expressed genes from the functional association network are further shown as volcano plots (Figure 6b). Notably, PARP9, PCSK6, TENM2, and PARP14 are upregulated, and RCAN2, TENM1, AR, RUNX1T1, LPAR1, EDIL3, MACROD1/2, ZEB2, PCSK2/5, CBFA2T2/3, RSPO1, and OARD1 are downregulated in lesional PN skin compared with control skin.

## 4. Discussion

Our genetic, transcriptomic, and population-based studies suggest that PN is strongly associated with a dysregulated skin–liver axis. Population-level analysis revealed higher odds of several hepatic complications in PN compared with control patients. PN was particularly associated with liver disease, liver fibrosis and cirrhosis, acute and subacute hepatic failure, inflammatory liver disease, nonalcoholic steatohepatitis, portal hypertension, fatty liver, chronic passive congestion, and HCC. Although pruritus is common in liver disease, few studies have examined the relationship between liver diseases and pruritus in depth. Pruritus affects 60–70% of patients with primary biliary cholangitis, 8% of patients with chronic hepatitis B, and 2.5–20% of patients with chronic hepatitis C [25,26,27]. Cholestasis due to hepatitis or cirrhosis has also been shown to cause itching, possibly related to increased bile [16,28]. Here, we highlight associations between PN and liver diseases that have not yet been described. Our findings suggest that there may be a different pathophysiology associated more specifically with PN and liver diseases. We aim to better understand the underlying mechanisms, while further investigating the recently recognized skin–liver axis. These findings are also relevant to clinical practice as they suggest that clinicians should have a lower threshold to screen for hepatic diseases in patients with PN.

RNA sequencing of PN skin biopsies further suggested that the cutaneous molecular signatures of PN patients overlap with those seen in liver disease. GSVA indicated the increased activation of gene sets associated with liver metabolism, chronic hepatic failure, acute hepatic failure, polycystic liver disease, cholestatic liver disease, and hepatocellular carcinoma. IPA of cutaneous gene expression in lesional PN skin indicated the significant activation of liver hyperplasia/proliferation, liver damage, liver inflammation/hepatitis, liver necrosis/cell death, liver cirrhosis, liver proliferation, increased ALP level, and increased LDH level pathways. An increased LDH level, often as a result of liver damage, is associated with fibrosis, and LDH has been found to strongly correlate with the clinical severity of inflammatory skin diseases like atopic dermatitis [29,30]. Elevated ALP levels may also be linked to pruritus by mechanisms like those seen in cholestasis [16].

To further explore the mechanisms underlying the skin–liver axis, we conducted a GWAS that revealed seven genes linked to SNPs overlapping in PN and liver disease. One of the identified genes was *AR*, which encodes the androgen receptor. *AR* has been shown to promote local inflammation through macrophage activation in the skin and plays an important role in HCC progression through activation of TGF-β1 expression in the liver [31,32,33]. TGF-β1 is important in wound healing as it promotes the proliferation and differentiation of dermal fibroblasts necessary for scar formation [34,35,36,37]. Dysregulation in TGF-β signaling may also lead to the silencing of *RUNX1* [38]. *RUNX1* expression is downregulated in psoriasis, and underexpression in the liver is a marker of metastasis [39,40,41,42]. Additionally, TGF-β1 signaling induces the expression of interleukin-31 (IL-31), a cytokine that is significantly upregulated in pruritic inflammatory skin conditions [37,43]. IL-31 has been associated with sensory neuronal outgrowth, activation of ion channels that transmit pruritic signals, and impaired keratinocyte differentiation that amplifies skin inflammation and itch [44,45,46]. Similarly to its role in dermal scar formation, the TGF-β1 pathway is a key component of liver fibrosis as it not only promotes fibroblast proliferation, but also drives the activation of hepatic stellate cells (HSCs) [47,48]. HSCs are the primary source of extracellular matrix substances that accumulate during fibrosis [47,48,49,50]. Consequently, the apoptosis of activated HSCs is important for fibrosis resolution [51]. *ZEB2*, another gene identified in the GWAS, has been suggested to interfere with this apoptosis and has increased expression in liver fibrosis [52]. In the skin, *ZEB2* has been linked with hyperproliferation of the epidermis and upregulation in keloid tissue [53,54]. *ZEB2* is also a well-known transcription factor inducing the epithelial-to-mesenchymal transition (EMT), where epithelial cells lose adhesive properties and assume a mesenchymal phenotype with enhanced migratory activity [54,55]. Although EMT is a pivotal process in development, it can also manifest in pathological conditions such as fibrosis, tumor invasion, and metastasis [56]. Other genes identified in our GWAS that are associated with EMT include *EDIL3* and *MACROD2*. *EDIL3* induces angiogenesis and sends pro-survival signals needed for EMT [57]. *EDIL3* has been shown to be overexpressed in psoriatic skin lesions and implicated in angiogenesis [58]. In the liver, *EDIL3* is elevated in HCC and higher expression is a prognostic factor for poor overall survival, possibly due to the overexpression of *EDIL3* triggering TGF-β signaling that further promotes angiogenesis and invasion [59,60,61]. Another predictor of poor prognosis in patients with HCC is the low expression of *MACROD2*, a DNA repair gene. Downregulation of *MACROD2* has been shown to increase the proliferation, invasiveness, and EMT phenotype of HCC cells [62]. In the skin, decreased expression of *MACROD2* has been associated with psoriatic lesions and hyperpigmented skin [63,64]. *TENM4* is also upregulated in liver fibrosis and contributes to cancer progression, although its role in the skin is unclear [65,66]. *PCSK5* is upregulated in liver fibrosis and may also be a biomarker reflecting local disease activity in inflammatory skin disease [67,68].

The epidemiologic, cutaneous, and population-level findings in this study suggest a strong association between PN and hepatic dysfunction, further supporting a role for dysregulation of the skin–liver axis in PN. GWAS identified genes involved in fibrosis, EMT, and angiogenesis. The dysregulation of these genes in both the liver and skin highlights TGF-β signaling as a potential mechanistic connection between the two pathologies. Given the significant burden of pruritus in liver diseases, further research is needed on the skin–liver axis to help guide clinical management of these associated conditions.

This study is limited by the small sample size and recruitment of patients from a single tertiary care center, restricting generalizability. Due to the cross-sectional design, conclusions cannot be drawn about causality and temporality. Additionally, patients in the epidemiologic study were not adjusted for hepatitis B virus carrier status, substance use, and other systemic comorbidities such as diabetes mellitus. Despite these limitations, we present novel findings highlighting the dysregulation of liver-disease-related pathways in PN that provide a basis for future studies interrogating the relationship between liver disease and PN. In addition, these findings may help identify future therapeutic targets and drive the development of much-needed treatments for PN.

## Data Availability

The FinnGen database, which consists of genotype data from Finnish biobanks and phenotype data from Finnish health registers, is available at https://www.finngen.fi/en (accessed on 16 September 2021).
